# Low Frequency Dielectric Relaxation and Conductance of Solid Polymer Electrolytes with PEO and Blends of PEO and PMMA

**DOI:** 10.3390/polym12051009

**Published:** 2020-04-27

**Authors:** Chin Han Chan, Hans-Werner Kammer

**Affiliations:** Faculty of Applied Sciences, University Teknologi MARA, Shah Alam 40500, Malaysia; hans-werner.kammer@jsk-medianet.de

**Keywords:** impedance spectra, dielectric response, polarization relaxation, scaled conductivity

## Abstract

Solid polymer electrolytes are mixtures of polymer and inorganic salt. There are quite a number of studies dealing with the relationship between electric conductivity and structural relaxation in solid polymer electrolytes. We present a phenomenological approach based on fluctuation-dissipation processes. Phase heterogeneity appears in poly(ethylene oxide) (PEO) of high molecular mass and its blends due to crystallization and accompanying phase segregation. Addition of salt hampers crystallization, causing dynamic heterogeneity of the salt mixtures. Conductivity is bound to amorphous phase; the conductivity mechanism does not depend on content of added salt. One observes dispersion of conductivity relaxation only at low frequency. This is also true for blends with poly(methyl methacrylate) (PMMA). In blends, the dynamics of relaxation depend on glass transition of the system. Glassy PMMA hampers relaxation at room temperature. Relaxation can only be observed when salt content is sufficiently high. As long as blends are in rubbery state at room temperature, they behave PEO-like. Blends turn into glassy state when PMMA is in excess. Decoupling of long-ranging and dielectric short-ranging relaxation can be observed. Conductivity mechanism in PEO, as well as in blends with PMMA were analyzed in terms of complex impedance Z*, complex permittivity, tangent loss spectra and complex conductivity.

## 1. Introduction

Poly(ethylene oxide) (PEO) is frequently used as a polymer matrix in polymer-salt mixtures. PEO exhibits low glass transition temperature and, consequently, sufficient chain flexibility at ambient temperature. Moreover, it possesses solubility of lithium salts. On the other hand, PEO is a semi-crystalline material. Its amorphous domains are intermingled with spherulites at room temperature. Thus, we have amorphous and spherulitic structures separated by inter-phase regions. Electric conductivity appears mainly in amorphous regions of neat or salt-comprising PEO, but might be influenced by the special semi-crystalline structure [1,2,3].

Properties of PEO are coined by hydrogen-bonding interactions in the amorphous state [4]. We may see it as associative polymer having the ability to form together with salt molecules reversible networks of physical bonds. It is important to note that weak physical bonds, as hydrogen bonds, develop and decay in experimental time scales of impedance spectroscopy. They are not necessarily stable during time scales of the experiment. These weak bonds are several times formed and broken during an experiment. 

It is obvious that cross-linked arrangement of chains develops during the process of sample preparation by solvent casting for impedance experiments. The action is characterized by a strong increase in viscosity and developing of elasticity. Moreover, the process leads to the phase morphology mentioned before.

Experiments have revealed that polymer-salt mixtures comprising PEO do not only consist of an amorphous network-like phase [5,6,7,8,9], but also of crystalline domains. Let us briefly summarize the essentials of phase morphology relevant to our discussion.

Below melting temperature of PEO, one may recognize two phases:

(I) Neat crystalline PEO
(II) Amorphous network phase with small amount of dissolved salt

Additionally, we have an interfacial region between these phases:

(I)–(II) Salt molecule-chain complexes interspersed between phases (I) and (II) forming an interfacial region between them and exhibiting different properties as phases (I) and (II).

Formation of reversible cross-links leads to attraction between chain molecules and rejection of salt. It may lead to phase separation—under non-equilibrium condition—into salt-polymer mixtures comprising different concentration of salt. But this is seen in integral way under relaxation appearing in impedance spectroscopy.

Understanding the nature of charge transport in solid polymer electrolytes proved no simple matter of concern. The amorphous phase of a salt comprising polymer is a rubbery liquid. One immediately recognizes that Frenkel’s [10] or Schottky’s [11] mechanisms of charge transport in solid polymer electrolytes do not apply here, because conductivity is bound to amorphous regions. Moreover, this phase is not in thermodynamic equilibrium. Therefore, it is adequate to consider mobility of relaxing processes in phase II) as fluctuation-dissipation process. In other words, we do not connect continuously scaled transport properties as electric conductivity to microscopic transport properties as ion diffusivities. In the following studies, continuum properties as conductivity were measured by impedance spectroscopy and related to relaxation in dielectric systems. 

Solid polymer electrolytes based on poly(ethylene oxide) (PEO) with lithium salt are prepared for use in advanced batteries. As mentioned above, highly flexible PEO chains comprising functional ether groups constitute a solvent with low driving force for solubility of inorganic salts. These mixtures exhibit low electric conductivity at ambient temperature, an effect caused by both constituents of the mixture. It is due to low density of charged entities participating in conduction process owing to poor arrangement of charged entities with respect to the electric field. In other words, it is caused by low entropy production under influence of the electric field and concomitantly by slow relaxations in amorphous phase. Additionally, crystallization of PEO restricts unfolding of the amorphous network phase, which means the establishment of in-homogeneities in the polymer-salt system.

Chiefly, the following routes have been pursued for enhancing conductivity in PEO-based systems at room temperature: 

Firstly, variation of salt concentration has been studied, Refs. [12,13,14,15,16]. 

Secondly, establishing of networks in the host polymer was performed by the cross-linking of low molecular PEO or the addition of ionic liquids, Refs. [17,18,19,20].

Thirdly, polymer blends with PEO have been probed [21]. Especially poly(methyl methacrylate) (PMMA) with its high tensile strength might be suitable to improve the mechanical stability of the host polymer. 

Fourthly, the host polymer is loaded with ceramic nanofillers for enhancement of conductivity by suppressing of crystallization [22,23,24,25,26]. 

In the following, we discuss the influence of constant salt concentration on dielectric properties of solid polymer electrolytes with PEO and blends with PMMA.

Conductivity of a solid polymer electrolyte is governed by shear rate given by ratio of drift velocity and diffusive length of network fluctuations; in other words, it is closely related to relaxation time constant ${\left(\omega_{\max}^{Z{}^{\prime \prime }}\right)}^{-1}$, which combines the effects of segmental motions and interactions with salt molecules or more generally, formation and breaking of weak physical bonds. Dependence of electric properties on temperature becomes obvious, but dependence on concentration of salt proves to be more complex. It turns out that the content of salt may influence alignment of charge carriers and chain segments under action of the electric field, and so also dynamic-mechanical properties of the polymer. Experiments suggest that dissolution of salt in the amorphous phase leads to dynamic mixture behaving ideally to good approximation. In that sense, content of added salt only indirectly affects electric properties. Transport properties are strongly influenced by network in the polymer. It is obvious that amorphous systems rich on hydrogen-bonding tend to formation of supra-molecular, network-like structures in liquid state. PEO may display similar behavior if it is transferred into amorphous rubbery state. Accordingly, dielectric behavior of weakly cross-linked PEO is ruled by delicately balanced entropic and energetic effects. Loading the polymer-salt system with ceramic nanofillers pursues the same goal as cross-linking, reduction of crystalline formations. It turns out that this procedure is less effective than cross-linking [26], owing to the strong tendency to agglomeration of nano-sized particles [27]. 

Electric properties of solid polymer electrolytes are studied by impedance spectroscopy. We cover the low-frequency range from 50 to 10^6^ Hz. This characterization of dielectric materials provides information about electrode polarization and electric relaxation processes. 

In the following, we will discuss blends of high molecular PEO and PMMA comprising the inorganic salt lithium perchlorate (LiClO_4_). This study was carried under variation of salt concentration at constant temperature. The mixture of PEO with the variation of Li-salt [15,16] serves as the reference for these blend system. 

## 2. Experimental

Sample preparation of solid polymer electrolytes consisting of PEO and LiClO_4_ and experimental procedures in impedance spectroscopy are published elsewhere [13]. In this section, we focus on similar procedures concerning blends of PEO and PMMA comprising LiClO_4_.

### 2.1. Materials

Both semi-crystalline PEO (Fischer Scientific Co., Fair Lawn, NJ, USA) with average molecular mass *M*_w_ = 300 kg mol^−1^, glass transition temperature *T*_g_ = −52 °C and melting temperature *T*_m_ = 65 °C and atactic PMMA (Sigma Aldrich Chemical Co., Saint Louis, MO, USA) with *M*_w_ = 350 kg mol^−1^ and *T*_g_ = 105 °C were purified by dissolution in solvent and precipitated with non-solvent. LiClO_4_ (Sigma Aldrich Chemical Co., Saint Louis, MO, USA) was dried in a conventional oven at 120 °C for 24 h before sample preparation. 

PEO and PMMA were dissolved in acetonitrile (Merck, Darmstadt, Germany) for preparation of 4 wt % polymer solution with blend compositions of PEO/PMMA from 1/0 to 0/1 in steps of 0.25. LiClO_4_ was added subsequently to the polymer solution. The polymer-salt mixture was stirred at 50 °C until the salt was completely dissolved. The viscous solution was slowly poured into the Teflon^©^ dish and dried slowly in a fume hood at room temperature. The thin film of the polymer-salt mixture was dried at 50 °C for 24 h in a conventional oven before it was heated up to 80 °C under a nitrogen atmosphere for 2 h for mixing of the polymer-salt in melt. Afterwards, the system was further dried in a vacuum oven for 48 h at 50 °C. The dried samples were stored in desiccators. Before characterization by impedance, samples were dried again in vacuum oven at 50 °C for 24 h. 

Salt concentration is defined by *Y*_S_ = *m*_salt_/*m*_poly_, with *m*_poly_ being mass of the blend under the discussion.

### 2.2. Differential Scanning Calorimetry (DSC)

Estimation of *T*_g_ was made from the heating cycle by the modulated DSC using TA Q2000-1509 (TA Instruments, New Castle, DE, USA). Before sample analysis, the DSC was calibrated using indium and sapphire standards. In order to minimize the thermo-oxidative degradation, nitrogen gas was purged throughout the analysis. Approximately, 10 mg of each sample was used. The sample was quenched by cooling to −90 °C for 5 min and was heated up to 120 °C at a scan rate of 2 °C min^−1^. The temperature modulation amplitude and period used were 1.27 °C and 60 s, respectively.

### 2.3. Impedance Spectroscopy (IS)

Impedances were determined at room temperature (25 °C) using a Hioki 3532-50 Hi Tester impedance analyzer (Hioki, Nagano-ken, Japan) over a frequency range from 50 Hz to 1MHz. Thin sample films were sandwiched between stainless steel electrodes. The electrode acts as current collector and blocking electrode. 

## 3. Results and Discussion

### 3.1. Glass Transition Temperature

**Figure 1:**
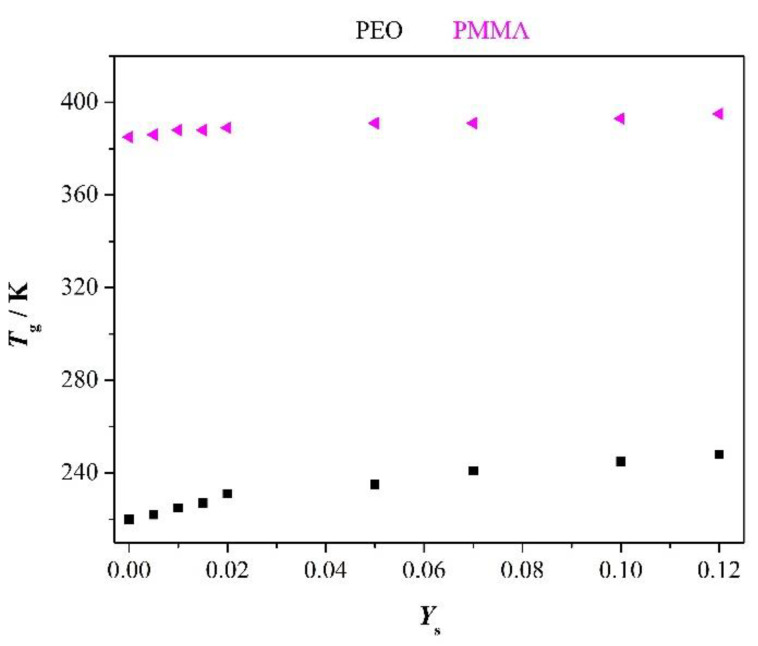
Glass transition temperature of the two parent polymers versus added salt content *Y*_S_.

We will discuss dielectric properties of polymer-salt mixtures at room temperature under variation of added Li-salt content. PEO as well as blends of it with PMMA serve as polymer host. We present in Figure 1, for orientation, glass transition temperature of the parent polymers versus salt content *Y*_s_ = *m*_salt_/*m*_poly_. The DSC scans are depicted in Figure S1 (Supplementary File). The point of interest, glass transition temperature *T*_g_ of the parent polymers, depends only weakly on salt content. Secondly, the amorphous rubbery state of PEO at room temperature is far above of its *T*_g_ whereas that one of PMMA is far below it. Thus, we have combination of a liquid-like and a glassy state in the blends. The essential point is that this behavior does not depend on salt distribution in the amorphous phase of the blends. Moreover, we note that the dielectric behavior of the blends is coined by the amorphous regions. Therefore, the detailed morphology of the systems is outside the scope in that context. 

### 3.2. Impedance Spectra of PEO

**Figure 2:**
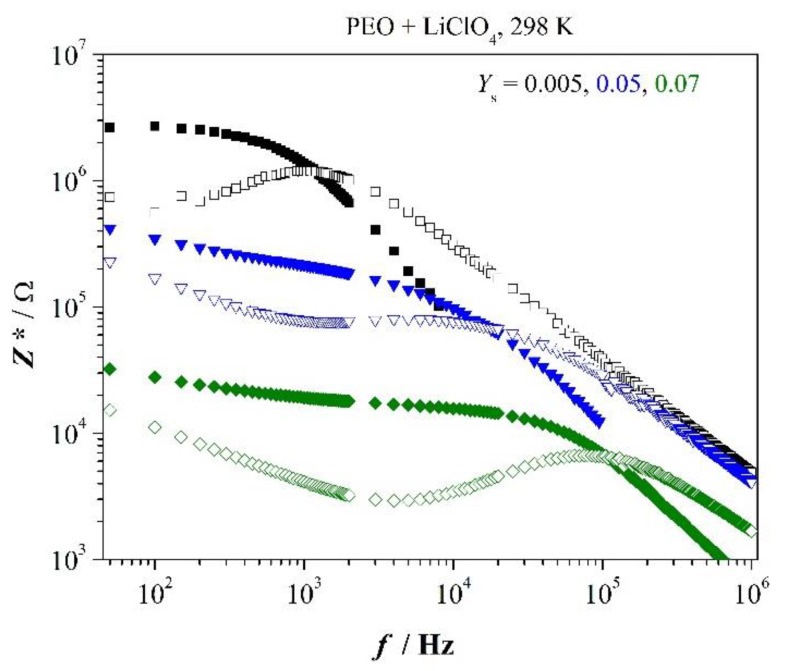
Impedance spectra of PEO with indicated salt concentration; *Z*″—open marker.

Figure 2 presents selected impedance spectra, *Z’*(*f*) and *Z*″(*f*), of PEO-films containing the indicated mass ratios *Y*_s_ of salt. 

Main relaxation peak is symbolized by $f_{\max}^{Z{}^{\prime \prime }}$; increase of capacity-related *Z*″ appears at $f_{\min}^{Z{}^{\prime \prime }}$. We recognize dielectric relaxation time ${\left(\omega_{\max}^{Z{}^{\prime \prime }}\right)}^{-1}$ as well as relaxation time ${\left(\omega_{\min}^{Z{}^{\prime \prime }}\right)}^{-1}$, which is close to longest relaxation time (see under tangent loss spectra), shortens with increasing salt content. Bulk resistance clearly decreases with increasing salt content which implies increasing dc conductivity. Moreover, one recognizes to good approximation at low concentration of salt

(1) $$f_{\max}^{Z{}^{\prime \prime }}=f_{cross}^{Z{}^{\prime }-Z{}^{\prime \prime }}$$

(1.1∙10^3^ Hz at *Y*_s_ = 0.005) whereas at higher concentration appears

(2) $$f_{\max}^{Z{}^{\prime \prime }}<f_{cross}^{Z{}^{\prime }-Z{}^{\prime \prime }}$$

(as an example, 7 × 10^3^ Hz < 1.3 × 10^4^ Hz at 0.05). It indicates the system follows Debye relaxation only at very low concentration of salt in the low-frequency region (refer Figure S2 in Supplementary File). Interaction between dipoles cannot be neglected at higher concentration of salt that is deviation appears from Debye relaxation or dispersion of relaxation frequencies is observed. Therefore, we see $f_{\max}^{Z{}^{\prime \prime }}$ as average over assemble of relaxation frequencies. 

Just for completeness, we list characteristic frequencies of the polymer-salt system in Appendix A, Table A1.

### 3.3. Impedance Spectra of PEO/PMMA Blends

**Figure 3:**
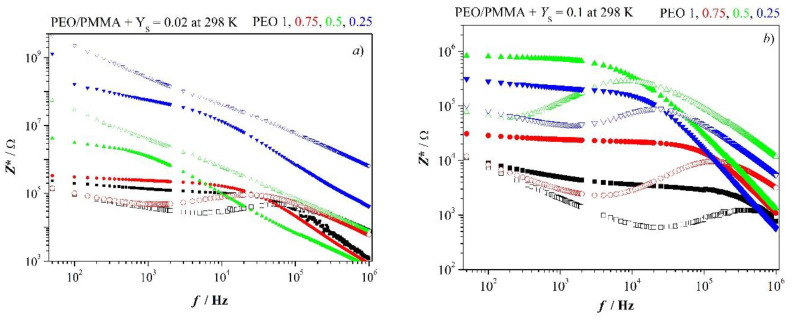
Impedance spectra for blends PEO/PMMA 1/0, 0.75/0.25, 0.5/0.5 and 0.25/0.75, *Z’* solid marker; (**a**) *Y*_S_ = 0.02, (**b**) *Y*_S_ = 0.1.

Figure 3 presents impedance spectra of PEO/PMMA blends containing low and high concentration of salt, respectively. PMMA is in the glassy state and consequently, one does not see any relaxation of the PMMA-salt mixture. On the other side, PEO displays nice dielectric relaxation. However, rigid PMMA prevents relaxation at low salt content already in symmetric blend (0.5/0.5) PEO/PMMA. Remarkable points of the spectra might be registered as follows. Blends with PEO in excess behave like neat PEO at low salt concentration. The amorphous network-like phase of PEO is almost not influenced by the salty PMMA phase. However, one registers that PEO phase contains slightly less salt than in the neat PEO; as a consequence, bulk resistance is slightly enhanced. Additionally, we note that the characteristic frequencies are shifted to lower values in the blend with PEO in excess as compared to neat PEO.

(3) $$f_{\min}^{Z{}^{\prime \prime }},f_{\max}^{Z{}^{\prime \prime }}\left(1/0\right)>f_{\min}^{Z{}^{\prime \prime }},f_{\max}^{Z{}^{\prime \prime }}\left(0.75/0.25\right)\;f_{cross}^{Z{}^{\prime }-Z{}^{\prime \prime }}\left(1/0\right)>f_{cross}^{Z{}^{\prime }-Z{}^{\prime \prime }}\left(0.75/0.25\right)$$

Dispersion of relaxation frequencies appears also in the blend with PEO in excess at low salt content. Retarding effects of the more viscous PMMA phase become increasingly influential with ascending PMMA content. Relaxation is suppressed at low salt concentration as Figure 3a shows. Let us summarize marked results for blends comprising low salt content. One observes inequality $f_{\max}^{Z{}^{\prime \prime }}<f_{cross}^{Z{}^{\prime \prime }-Z{}^{\prime }}$ for blends 1/0 and 0.75/0.25. 

It tells us that dielectric relaxation in both electrolytes deviates from Debye-like relaxation. They are characterized by dispersion of relaxation times. The average relaxation time of PEO with *Y*_S_ = 0.02 amounts to 3 × 10^−6^ s. It doubles when the mixture of the two parent polymers comprises 25% of PMMA. Addition of higher relative amounts of PMMA leads to infinite average relaxation time. 

The situation changes with high salt concentration, Figure 3b. Enhanced content of PMMA in blends leads increasingly to higher bulk resistance at *Y*_S_ = *const*, simultaneously, characteristic frequencies shift to lower values. It indicates salt content of the amorphous PEO phase lessens more and more. Again, one observes dispersion of relaxation times. But there is an exception from this curve. The blend with excess of PMMA breaks the trend sketched before. Bulk resistance decreases and characteristic frequencies increase. 

Just for completeness, we list characteristic frequencies of the polymer-salt system with *Y*_S_ = 0.1 in Appendix A, Table A2. It also includes permittivity, polarization and longest relaxation time ${\left(\omega_{\max}^{\tan\delta }\right)}^{-1}$ as well as conductance characteristics for later comparison. 

The amorphous salt comprising phase of PMMA supports relaxation of PEO phase by retarding crystallization of PEO. Glass transition temperatures of the parent polymers with *Y*_S_ = 0.1 read after Figure 1 *T*_g_ (PEO) ≈ 220 K and *T*_g_ (PMMA) ≈ 390 K. If one assumes, the two constituents are miscible in the amorphous state [28] and the *T*_g_s follow Fox equation, then we get for the 50/50 blend *T*_g_ ≈ 8 °C and for the 25/75 blend *T*_g_ ≈ 54 °C. It follows the 50/50 blend is at room temperature above glass transition temperature (as the 75/25 blend). The blend behaves accordingly, as given in Figure 3b. The situation changes with the blend having PMMA in excess. Under conditions of miscibility, the system is below *T*_g_ at room temperature. As a result, we have tendency to decoupling of structural and electric relaxation due to frozen in segmental relaxation. We are coming back to this important point in the next paragraph. 

### 3.4. Relaxation after Impedance and Electric Modulus Spectra

Relaxations might be characterized by imaginary parts of electric modulus and impedance. 

Both are closely related to dynamic quantities

(4) $$M{}^{\prime \prime }\propto \dot{Z}{}^{\prime }\;\mathrm{and}\;Z{}^{\prime \prime }\propto \dot{\varepsilon }{}^{\prime }$$

Accordingly, *M*″ records long-ranging electric or non-local relaxation whereas *Z*″ reflects trap-controlled or local relaxation. More explicitly, we have instead of Eqs. (4)

*M*″ = *ωZ’C*_o_ and *Z*″ = *ωε′C*_o_|*Z*|^2^ (5)

Quantity *C*_o_ represents the capacity of sample geometry, *C*_o_ = *ε*_o_*A/*2*d* (*A*—area of the electrode, *2d*—sample thickness). It becomes obvious that coincidence of scaled functions *Z”/Z*_max_”(*f*) and *M”/M*_max_”(*f*) indicates long-ranging electric relaxation whereas mismatch of the two functions suggests dielectric or local relaxation.

**Figure 4:**
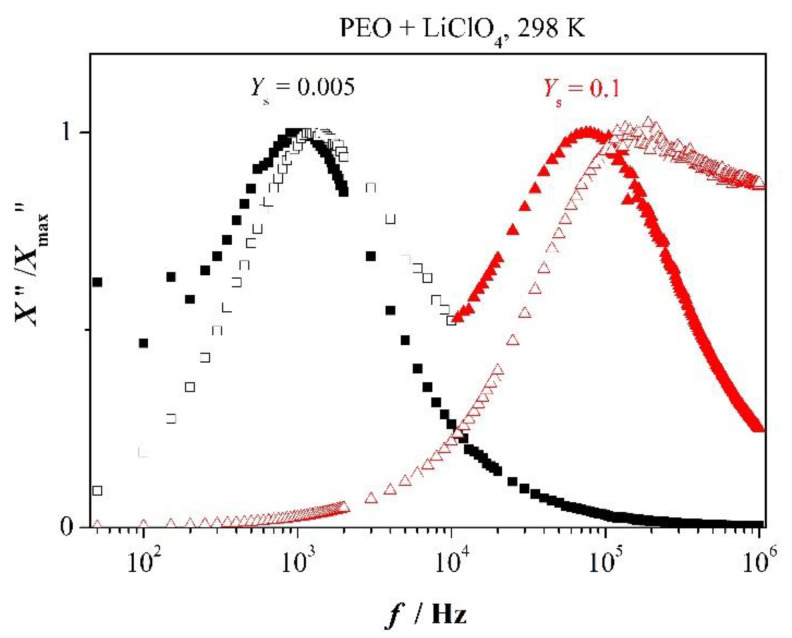
Scaled imaginary parts of impedance Z* and electric modulus M*; scaled *Z*″—solid marker, scaled *M*″—open marker.

Debye relaxation and deviation from it are nicely illustrated in Figure 4 by comparison of the scaled functions of PEO with different salt content in the low-frequency range

(6) $$\omega_{\min}^{Z{}^{\prime \prime }}\leq \omega \leq \omega_{\max}^{Z{}^{\prime \prime }}$$

One clearly recognizes that the two functions coincide to acceptable approximation for the lowest concentration of added salt but do not so for higher salt content. It demonstrates that the corresponding peak in Figure 4 reflects prevalence of long-range or non-local motion of charged entities at low concentration whereas supremacy of localized or trap-controlled relaxation appears at higher concentration. This behavior is in agreement with inequality (2).

**Figure 5:**
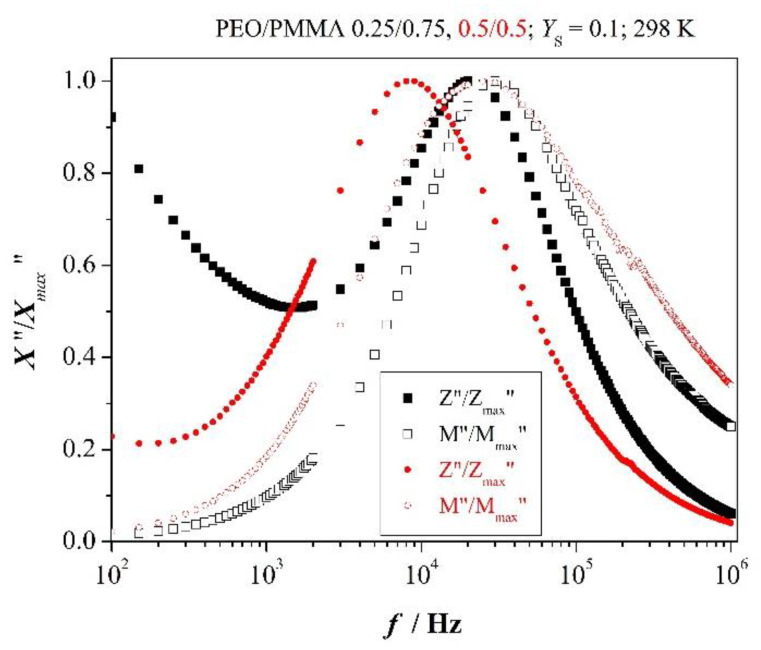
Scaled imaginary parts of impedance Z* and electric modulus M*; scaled *Z*″—solid marker, scaled *M*″—open marker.

Perception is similar for blends with high salt content as given in Figure 5. Scaled *Z*″ and *M*″ are almost matching for the blend with PMMA in excess, but not so for the symmetric blend. Moreover, the width of half-maxima of *M*″ spectra is greater than 1.144 decades. This indicates deviation from Debye-like relaxation and distribution of relaxation times. It is also nicely confirmed by shortening of relaxation times ${\left(\omega_{\max}^{Z{}^{\prime \prime }}\right)}^{-1}$ as presented in Figure 3b. They amount to 1.8 × 10^−5^ s for the symmetric blend, but shorten to 8 × 10^−6^ s with PMMA in excess. Dynamics of relaxation is further detailed with Equation (27) below.

### 3.5. Permittivity Spectra of PEO

Complex permittivity spectra are as important as impedance spectra of Figure 2. This is so because conductivity is proportional to dynamic permittivity under periodic conditions, σ* ∝ $\dot{\varepsilon }*$. It follows

σ* = *iωε*_o_ε* (7)

**Figure 6:**
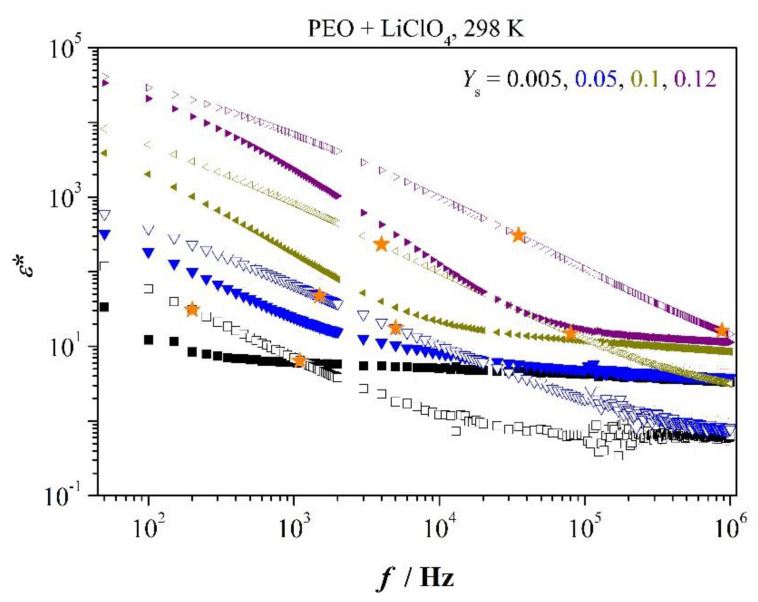
Selected permittivity spectra for the indicated salt concentration; *ε′*—solid marker; stars mark the low-frequency range $f_{\min}^{Z{}^{\prime \prime }}$…$f_{\max}^{Z{}^{\prime \prime }}$.

Permittivity spectra for neat PEO are presented in Figure 6. The low-frequency range, Equation (6), is marked by stars. As can be recognized by optical inspection, dielectric loss *ε*″ displays power-law dependence on frequency in that range

(8) $$\varepsilon {}^{\prime \prime }={\left(\frac{\omega^{+}}{\omega }\right)}^{n}\;\mathrm{with}\;n\leq \;1$$

Moreover, one observes analogously to Equations (1) and (2) at low concentration of salt

(9) $$f_{cross}^{\varepsilon {}^{\prime }-\varepsilon {}^{\prime \prime }}=f_{\max}^{Z{}^{\prime \prime }}$$

and at higher concentration

(10) $$f_{cross}^{\varepsilon {}^{\prime }-\varepsilon {}^{\prime \prime }}>f_{\max}^{Z{}^{\prime \prime }}$$

(refer Figure S3 in Supplementary File).

Function *ε*″(*f*) is closely related to dc conductivity in low-frequency range following Equation (7). Power-law dependence of *ε*″ determines the weak dependence of *σ*_dc_ on frequency. It follows with Equation (8)

*σ*_dc_ = *ε*_o_ (*ω*^+^)^n^*ω*^1−n^ (11)

Exponent *n* (refer to Table A1) displays quite interesting variation. It approaches to good approximation Debye-like relaxation at low concentration whereas it becomes distant to it at higher concentration. This behavior is completely equivalent to distance of frequencies $f_{\max}^{Z{}^{\prime \prime }}$ and $f_{cross}^{\varepsilon {}^{\prime }-\varepsilon {}^{\prime \prime }}$ in Figure 6. Mismatching of scaled quantities *Z*″ and *M*″ points to the same circumstance in Figure 4. The mixture with *Y*_s_ = 0.05 displays major deviation of exponent *n* from unity and equivalently, the highest distance between the two characteristic frequencies appears in the very mixture, as shown in Figure 6. As a result, dc conductivity is quite low for this concentration, indicating only a low amount of salt is dissolved in the network-like amorphous phase. Perhaps, it is related to sample preparation. We note criterions (8) and (10) for deviation from Debye-like relaxation are equivalent. Characteristic frequency $f^{+}$ increases monotonously with concentration indicating descending resistance and ascending capacity of the system, but generating a dent in conductivity at *Y*_S_ = 0.05. Distance of exponent *n* from unity is also indicative of dispersion of relaxation-time constants. Relevant parameters to permittivity are summarized in Appendix A, Table A1.

### 3.6. Permittivity Spectra of Blends

**Figure 7:**
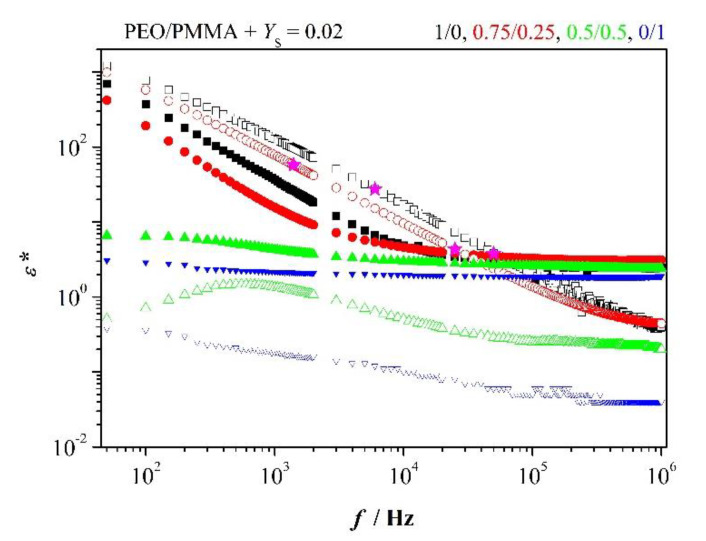
Permittivity spectra for the indicated blends and salt content; stars manifest range (6).

Blends, comprising low content of salt, display relaxation only when PEO is in excess. This is exemplified in Figure 7. Relevant parameters are inserted in the figure. One recognizes addition of 25 % PMMA reduces slightly polarization and only to minor extent conductivity at $f_{\max}^{Z{}^{\prime \prime }}$. Moreover, relaxation is completely suppressed when PMMA turns to excess.

Sufficiently high content of salt leads in all blends to relaxation as Figure 3b reveals. We focus on blends with sufficiently high salt content added, *Y*_S_ = 0.1. Similar graphs on permittivity as in Figure 6 evolve. Parameters reflect basically the same behavior as discussed for impedance spectra of blends. Here, it is manifested in frequency *f*^+^, decreasing successively with increasing PMMA content as long as PEO is in excess. However, limiting frequency increases slightly when PMMA turns to major constituent. Conductivity behaves concomitantly with increasing PMMA. Adding PMMA in steps of 0.25 to PEO reduces conductivity.

*ε′*—solid marker; relevant data for the blends 1/0 and 0.75/0.25:

*f*^+^ = 2.2 × 10^5^ Hz, *n* = 0.92, *σ*_dc_ = 9.4 × 10^−6^ A/(V m); *f*^+^ = 1.3 × 10^5^ Hz, *n* = 0.90, *σ*_dc_ = 5.5 × 10^−6^ A/(V m)

But this is only true as long as PMMA is not in excess. If it is so, one observes the increase in conductivity as discussed before. Enhanced conductivity and frequency $f^{+}$ are in accordance with reduced polarization relaxation. Parameters are listed in Appendix A, Table A2.

We note also

(12) $$f^{+}>\;f_{cross}^{\varepsilon {}^{\prime }-\varepsilon {}^{\prime \prime }}$$

This is so because *ε*″($f_{cross}^{\varepsilon {}^{\prime }-\varepsilon {}^{\prime \prime }}$) > 1.

### 3.7. Tangent Loss Spectra of PEO and Blends PEO/PMMA

After having chiefly circled around $\omega_{\max}^{Z{}^{\prime \prime }}$, we turn to the lower characteristic frequency $\omega_{\min}^{Z{}^{\prime \prime }}$. The tangent loss function plays the part analogous to imaginary part of electric modulus with respect to frequency $\omega_{\max}^{Z{}^{\prime \prime }}$; the peak of tangent-loss spectrum is situated at frequency $f_{\max}^{\tan\delta }$. Inverse of this frequency might be seen as longest relaxation time. Analog to equality Equation (1), one observes under Debye-like relaxation the following equality

(13) $$f_{\min}^{Z{}^{\prime \prime }}=f_{\max}^{\tan\delta }$$

For polarization relaxation outside Debye-like relaxation, one finds instead of Equation (13)

(14) $$f_{\max}^{\tan\delta }<f_{\min}^{Z{}^{\prime \prime }}$$

**Figure 8:**
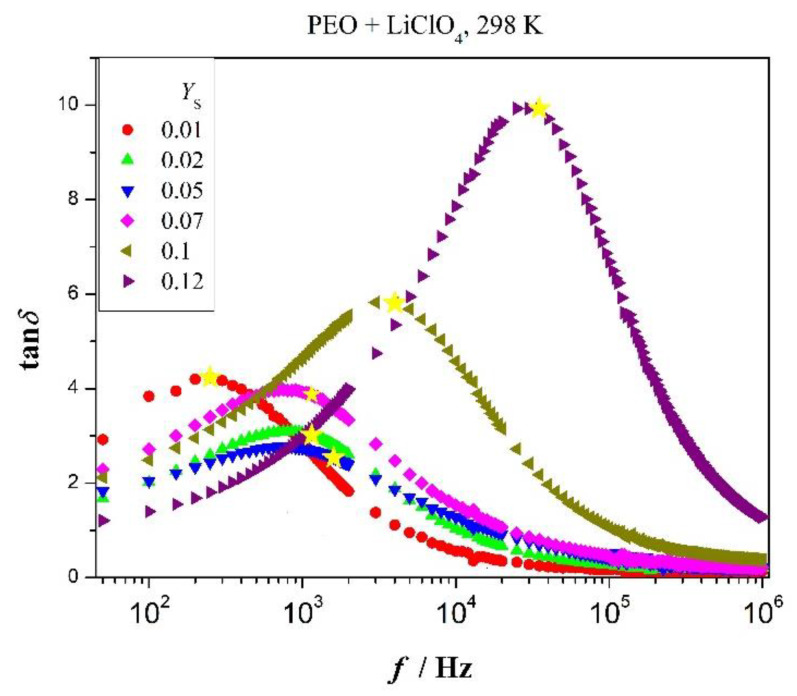
Tangent-loss spectra for *T* = *const* and indicated concentration of added salt as parameter; stars mark characteristic frequency $f_{\min}^{Z{}^{\prime \prime }}$.

A few tangent-loss spectra of PEO are presented in Figure 8. They refer to added relative amount of LiClO_4_ as varying parameter and *T* = *const*. One observes Debye-like relaxation, consistent with equality (13), to good approximation at low concentration of added salt. Deviation from Debye-like relaxation appears at higher concentration in agreement with Figure 2 and Table A1, that is the inequality (14) holds true. One observes irregular variation of tan*δ* (*f*) at *f* =$f_{\max}^{\tan\delta }$. It decreases at low salt content and increases at high salt concentration. The effect is quantitatively ruled by the quotient *ε”/ε′*($f_{\max}^{\tan\delta }$). Optical inspection of Figure 6 clearly confirms variation of (tan*δ*)_max_ shown in Figure 8. The dent between *Y*_S_ = 0.02 and 0.07 might be caused by balance between dissolution of salt in the amorphous phase and existence in interfacial region between crystalline and network phase. In that sense, Figure 8 reflects in-homogeneity of salt distribution in the salt-polymer mixture.

Let us sketch the physical meaning of peak height (tan*δ*)_max_. Peak intensity turns out to be inversely proportional to capacity ratio *A*_C_ = *λ/d* (2*d* − sample thickness) [29]. Length *λ* is associated with network displacement fluctuations. We approximate tangent loss peak (tan*δ*)_max_ as follows [30]:

(15) $$Z{}^{\prime \prime }=\omega \tau Z{}^{\prime }+\frac{R_{b}A_{C}}{\omega \tau }$$

.

Symbols mean: Average relaxation time *τ* = ($\omega_{\max}^{Z{}^{\prime \prime }}$)^−1^, *R*_b_—bulk resistance of the sample and capacity ratio *A*_C_. One easily shows [30] that *A*_C_ reads in simplest approximation

(16) $$A_{C}={\left(\frac{\omega_{\min}^{Z{}^{\prime \prime }}}{\omega_{\max}^{Z{}^{\prime \prime }}}\right)}^{2}$$

It follows from Equation (15)

(17) $$A_{C}={\left[{\left(\tan\delta \right)}_{\max}\left\{\left(\frac{\omega_{\max}^{\tan\delta }}{\omega_{\min}^{Z{}^{\prime \prime }}}\right)+{\left(\frac{\omega_{\max}^{\tan\delta }}{\omega_{\min}^{Z{}^{\prime \prime }}}\right)}^{-1}\right\}\right]}^{-2}$$

Fluctuation length *λ* is governed by polarization relaxation.

Inspection of Figure 8 shows polarization relaxation time and fluctuation length *λ* are roughly constant in concentration range 0.02 to 0.07. Length *λ* is inversely proportional to intensity of peak in tangent-loss spectra. The lower intensity the more extended length *λ*, the lower polarization. Thus, we may see intensity of tangent-loss peak as direct measure of polarization. However, peak height is coined by network displacement fluctuations that depend also on homogeneity of salt distribution. Increasing quantity (tan*δ*)_max_ with concentration of added salt signalizes descending double-layer thickness and concomitantly points to increase of friction or restoring energy during relaxation process at *T* = *const*. This is observed in Figure 8 at sufficiently high concentration. It is not seen for samples with 2 to 7 wt% of added salt. Obviously, these samples are quite heterogeneous or are coined by low solubility of salt in the amorphous PEO phase. Therefore, length *λ* is quite extended or in other words we do not observe Debye-like relaxation in that range, but dispersion of relaxation times. This is in agreement with permittivity curves in Figure 6. Also frequency $\omega_{\max}^{\tan\delta }$ stays approximately constant with added salt content in that range.

Relationship between capacity ratio *A*_C_ and (tan*δ*)_max_ characterizes polarization relaxation. Replacing *λ* by Stokes-Einstein relationship in Equation (17) yields for peak maximum in tangent loss spectra [30]

(18) $${\left(\tan\delta \right)}_{\max}^{2}\propto \frac{friction\;energy}{thermal\;energy}$$

Friction energy means actually the work done by restoring force during polarization relaxation over distance *λ* or just electric field energy dissipated in the resistance-capacity circuit. Squared peak intensity is directly proportional to restoring energy when thermal energy is kept constant.

**Figure 9:**
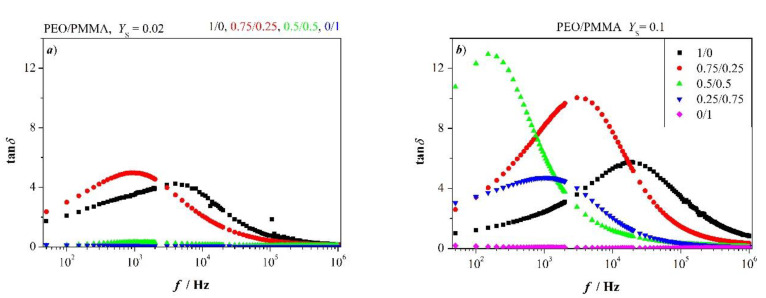
Tangent loss spectra for PEO/PMMA blends as indicated; (**a**) *Y*_S_ = 0.02, (**b**) *Y*_S_ = 0.1.

Tangent loss spectra of blends are shown in Figure 9 for two added salt concentrations. Graphs are striking, increase of (tan*δ*)_max_ appears conversely with respect to frequency in Figure 9 as compared to Figure 8.

Addition of the glassy PMMA to PEO increases (tan*δ*)_max_ and shifts frequency $f_{\max}^{\tan\delta }$ to lower value. This is true for low and high concentration of added salt; however, effects are more pronounced at a higher concentration of added salt. As a result, the addition of PMMA leads to prolonged longest relaxation time and to shrinking of fluctuation length *λ*. This happens as long as PMMA is not in excess. One observes slightly accelerated relaxation due to less delayed longest relaxation time and broader fluctuation length in the blend with PMMA in excess.

### 3.8. Conductivity and Conductivity Relaxation

**Figure 10:**
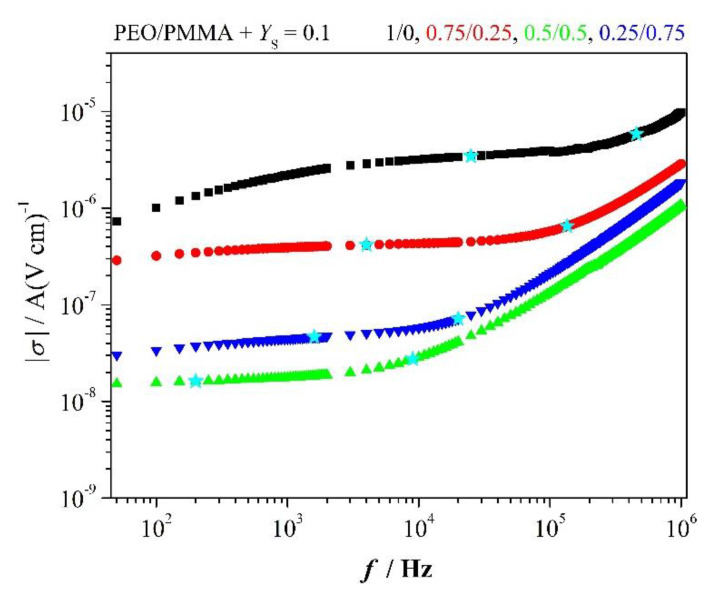
Conductivity spectra; stars indicate frequencies $f_{\min}^{Z{}^{\prime \prime }}$ and $f_{\max}^{Z{}^{\prime \prime }}$

Figure 10 presents conductivity spectra |*σ*| of selected PEO/PMMA blends comprising *Y*_S_ = 0.1 of LiClO_4_. Spectra |*σ*|(*f*) exhibit dc conductivity plateau, which drops and also shifts to lower frequency with increasing PMMA content, sandwiched between characteristic frequencies $f_{\min}^{Z{}^{\prime \prime }}$ and $f_{\max}^{Z{}^{\prime \prime }}$ for real part *σ’* of conductivity. Deviation from plateau occurs at low frequencies due to electrode polarization and at frequencies beyond $f_{\max}^{Z{}^{\prime \prime }}$ caused by trap-controlled dielectric relaxations. The slope of plateau is chiefly ruled by *ωε*″(*ω*) after Equation (8).

We note also that dc conductivity increases when PMMA is in excess, which is in accord with Figure 3b.

Conductivity normalized with respect to dc-conductivity, *σ/σ*_dc_, is seen as function of reduced frequency *f/f*_σ_. Frequency *f*_σ_ represents conductivity relaxation frequency. It might be related to bulk properties as proposed previously [30]:

(19) $$f_{\sigma }=\frac{\sigma_{dc}}{\pi \varepsilon_{o}\varepsilon_{\infty }}$$

Quantity *ε*_∞_ represents permittivity in the limit *f* → ∞. We immediately recognize that conductivity relaxation frequency is proportional to limiting frequency *f*^+^ of polarization relaxation in case of Debye relaxation

(20) $$f_{\sigma }\propto \frac{f^{+}}{\varepsilon_{\infty }}\;\mathrm{for}\;n=1$$

Equation (20) shows $f_{\sigma }<f^{+}$. Relaxation time *τ*_σ_ after Equation (19) and longest relaxation time, related to $f_{\max}^{\tan\delta }$, vary for the systems under discussion with added salt concentration at *T* = *const*. Dependence of relaxation on salt concentration becomes more transparent when one plots scaled conductivity versus reduced frequency *f*/*f*_σ_. In other words, it is of interest to elucidate to what proportion electric and structural relaxations contribute to conductivity. Scaling of conductivity may help in discussing this issue.

The longest relaxation time of PEO is almost two orders of magnitude longer as the relaxation time of conductivity. This effect is clearly more extended in blends with PMMA (cf. Table A2).

Reduced conductivity equals inverse reduced impedance:

(21) $$\frac{\left|\sigma \right|}{\sigma_{dc}}=\frac{2R_{b}}{\left|Z\right|}$$

Scaled function:

|*σ*|*/σ*_dc_ = g(*f/f*_σ_) (22)

elucidates whether the conductivity mechanism depends on salt content. Experimental results according to function (22) are presented in Figure 11. They reveal that the master curve after Equation (22) follows a power law at high frequency as given by

|*σ*|*/σ*_dc_ ∝ (*f/f*_σ_)^n^ (23)

Exponent *n* deviates from unity as it is manifested in Figure 11. It points to dominance of short-ranging dielectric relaxation. Figure 11a is not surprising; one expects conductivity mechanism being independent of added salt. Merely, weak dispersion might be observed at low frequency.

Strikingly, one observes PEO-like behavior also in blends with PMMA at sufficiently high *Y*_S_ = *const*. Thus, the blends are dynamically heterogeneous after Figure 3b and Figure 9b when PMMA is in excess, but the conductivity mechanism remains untouched.

Dispersion is ruled by polarization effects at low frequencies that turn out to be dependent on concentration. Conductivity spectra meet also in a master curve at very high frequencies. It indicates relaxation is coined by short-range motion of charged entities which does not depend on the concentration of salt. This is especially noticeable in the blend with PMMA in excess. Overlapping of curves appears even at a low frequency.

### 3.9. Remarks to Dynamics

As outlined above, friction energy is actually the work done by restoring force during polarization relaxation. Squared peak intensity is directly proportional to restoring energy when thermal energy is kept constant. Friction or restoring force during polarization relaxation is given after Stokes-Einstein relationship by

(24) $$D\eta =\frac{k_{B}T}{6\pi \lambda }$$

Equation (24) presents the force ruling relaxation of concentration fluctuation. In other words, it drives relaxation of network displacement fluctuation during polarization. This force is solely proportional to squared peak height (tan*δ*)_max_ for *T* = *const* as given by Equation (18). We approximate dynamics of restoring by assuming that the amorphous network phase forms a perfect salt-polymer mixture

*ηω* = *cRT* (25)

Quantity *c* represents density of molecules dissolved in the amorphous phase. It is given by

(26) $$c=\frac{\rho_{poly}}{M_{S}}Y_{S}^{amorphous}$$

where $Y_{S}^{amorphous}$ represents the concentration of salt dissolved in the amorphous phase. It is a tiny fraction of added salt, $Y_{S}^{amorphous}$/*Y*_S_ << 1. Therefore, approximation (25) is acceptable. The rate of restoring force follows by multiplying relationship (25) with diffusion coefficient *D* and replacing *Dc* by dc conductivity. It reads

(27) $$D\eta \omega =\sigma_{dc}{\left(\frac{RT}{F}\right)}^{2}$$

We get the nice result of Equation (27); the rate of restoring force is proportional to dc conductivity in low-frequency range.

**Figure 12:**
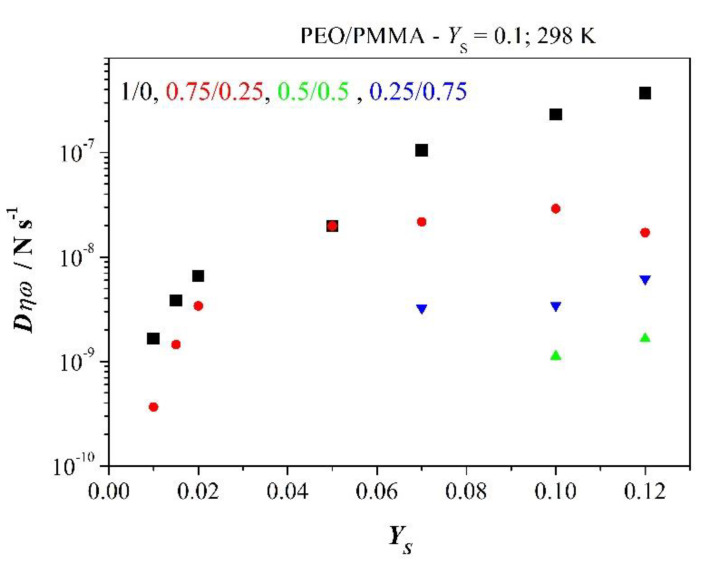
Force rate *Dηω* versus concentration of added salt *Y*_S._

Experimental results for PEO and blends with PMMA are plotted in Figure 12 as a function of added salt concentration *Y*_S_. The rate increases with concentration *Y*_S_ for PEO and tends to saturation which is given by solubility limit of salt in the amorphous phase of PEO and corresponding phase in blends. Solubility limit $c_{\lim}/c_{o}$ might be estimated from

(28) $$\frac{c_{\lim}}{c_{o}}={\left(\sigma_{dc}\right)}_{saturat}\frac{RT}{F^{2}c_{o}D}$$

Molar concentration *c*_o_ is the concentration of added salt after Equation (26), here with *Y*_S_ = 0.1 corresponding to Figure 12. Density of polymer is assumed to be *ρ* = 1 g/cm^3^ and drift velocity is approximated by *v*_drift_ = $\omega_{\max}^{Z{}^{\prime \prime }}$*λ*.

We may also estimate entropy production *P*. One gets for dissipated energy analogous to Equation (27).

(29) $$T\dot{S}=D\eta \omega \lambda$$

It follows for molar entropy production

(30) $$\frac{P}{R}=\sigma_{dc}\lambda \frac{RT}{Fe}$$

Relevant data to dynamics are listed in Table 1.

Behaviour discussed here is in complete agreement with spectra presented in Figure 5 showing matching of scaled functions *Z*″ and *M*″. Limiting value of dc conductivity or saturation descends with an increasing amount of PMMA in the blend, and so too behaves drift velocity. Addition of PMMA remarkably suppresses relaxation. It is reflected by weak entropy production in Table 1 or weak structure formation in the blend. But ratio *c*/*c*_o_ stays roughly constant pointing towards PEO-like behaviour of the phase. This respond might be observed as long as the blend remains above *T*_g_. The blend with PMMA in excess stays at room temperature in a glassy state. Thus, limiting solubility *c*_lim_/*c*_o_ decreases somewhat, however drift velocity increases due to the decoupling of electric and structural relaxation.

## 4. Conclusions

Dielectric behavior of PEO and of the blends with PMMA is governed by amorphous network-like phases. Addition of Li-salt leads to dissolution of tiny amounts of salt in these phases. Therefore, corresponding polymer-salt mixture can be seen to excellent approximation as a perfect solution. This behavior is also maintained when glassy PMMA is added to PEO at room temperature. One may see this as PEO-like behavior. High-molecular PEO and blends with PMMA display a nearly frequency-independent plateau of real impedance *Z’* at *T* = *const* and constant concentration of added salt. The plateau descends with increasing parameter *Y*_S_, salt content. Main relaxation peak shifts to higher frequency with growing on concentration of salt. In blends at room temperature, relaxation can be observed only at sufficiently high concentration of added Li-salt. The glassy PMMA hampers relaxation at low content of added salt. Conductivity relaxation time decreases by two orders of magnitude in PEO in the concentration range under discussion whereas it increases only slightly at *Y_S_* = 0.1 when PMMA is added.

Condensation of scaled conductivity of PEO in one master curve reveals that conductivity mechanism does not depend on added salt concentration. Also, conductivities of blends at *Y*_S_ = 0.1 overlap to one curve which manifests that conductivity mechanism is independent of blend composition. However, PEO and blends exhibit dispersion of conductivity at a low frequency associated with polarization relaxation.

Dynamics of relaxation in PEO/PMMA is quite challenging. Data of Table 1 tell us that fraction of dissolved salt in PEO-like phase stays roughly constant when PMMA is added. However, drift velocity as well as entropy production decrease strongly as long as the blend stays in the rubber-like state at room temperature. The situation changes when PMMA is in excess that is, the system turns in the glassy state. Suddenly, drift velocity slightly increases which points towards decoupling of electric and structural relaxation, decoupling is manifested by matching of scaled functions *M*” and *Z*” to acceptable approximation.

## Figures and Tables

**Figure 11:**
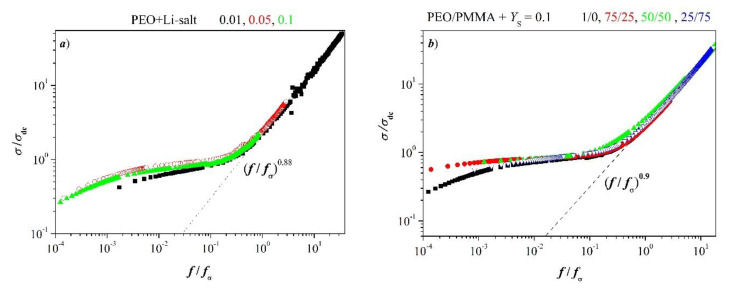
Scaled conductivity versus reduced frequency; power symbols stand for relationship (23), (**a**) PEO plus LiClO_4_ with indicated concentration of salt at *T* = 298 K, (**b**) Blends PEO/PMMA with *Y*_S_ = 0.1 for indicated blend composition at *T* = 298 K.

**Table 1:** About dynamics in PEO/PMMA blends with *Y*_S_ = 0.1 Li-salt at 298 K. Generalized diffusion coefficient, limiting solubility *c*_lim_*/c*_o_, drift velocity and entropy production.

	1/0	0.75/0.25	0.5/0.5	0.25/0.75
***DX**/*m^2^ s^−^^1^	9.9 × 10^−^^14^	1.3 × 10^−^^14^	4.8 × 10^−^^16^	1.5 × 10^−^^15^
***c*_lim_**/*c*_o_	1.2 × 10^−^^7^	7.3 × 10^−^^7^	6.5 × 10^−^^7^	3.1 × 10^−^^8^
***v*_drift_**/m s^−^^1^	3.96	0.30	0.016	0.19
***PR*^−1^**/10^7^·s^−^^1^	1.17	0.29	0.0095	0.13
***σ*_dc_**/A(V m)^−1^	5.6 × 10^−5^	5.1 × 10^−5^	2.1 × 10^−6^	5.4 × 10^−6^

## Supplementary Materials

The following are available online at https://www.mdpi.com/2073-4360/12/5/1009/s1, Figure S1: DSC scans for the two parent polymers versus added salt content γ_S_. Arrows show the glass transition temperatures, Figure S2: Impedance spectra of PEO with indicated salt concentration; Z”—open marker, Figure S3: Selected permittivity spectra for the indicated salt concentration; ε′—solid marker; stars mark the low-frequency range $f_{\min}^{Z{}^{\prime \prime }}$…$f_{\max}^{Z{}^{\prime \prime }}$.

## Appendix A

**Table A1:** Characteristic frequencies in system PEO + LiClO_4_ at 25 °C

*Y*_s_	0.01	0.02	0.05	0.07	0.1	0.12
$f_{\max}^{Z{}^{\prime \prime }}$/Hz	5000	5.0 × 10^4^	6.0 × 10^4^	1 × 10^5^	8.0 × 10^4^	6.3 × 10^5^
$f_{\min}^{Z{}^{\prime \prime }}$/Hz	250	1200	6000	8000	4000	3.5 × 10^4^
$f_{cross}^{Z{}^{\prime }-Z{}^{\prime \prime }}$/Hz	5 × 10^3^	7 × 10^4^	1.3 × 10^4^	1.3 × 10^5^	7.45 × 10^5^	>10^6^
*f*^+^ /Hz	2.7 × 10^4^	2.2 × 10^5^	1.5 × 10^5^	2.4 × 10^5^	1.5 × 10^6^	1.4 × 10^7^
*n*	0.93	0.92	0.83	0.87	0.92	0.96
$f_{\max}^{\tan\delta }$/Hz	250	750	750	950	3000	2.5 × 10^4^
${\left(\tan\delta \right)}_{\max}$	4.9	4.2	3.1	2.8	3.9	5.8
*σ**’/*A(V m)^−^^1^ at $f_{\max}^{Z{}^{\prime \prime }}$	7.4 × 10^−8^	2.4 × 10^−7^	9.6 × 10^−7^	1.1 × 10^−7^	5.6 × 10^−5^	1.2 × 10^−4^

**Table A2:** Characteristic quantities of PEO/PMMA blends with *Y*_S_ = 0.1 at 298 K

*Y*_s_	1/0	0.75/0.25	0.5/0.5	0.25/0.75
$f_{\max}^{Z{}^{\prime \prime }}$/s^−1^	8 × 10^4^	1.35 × 10^5^	9000	2.0 × 10^4^
$f_{\min}^{Z{}^{\prime \prime }}$/s^−1^	4000	4000	200	1600
*f*^+^/s^−1^	1.5 × 10^6^	1.0 × 10^6^	4.1 × 10^4^	1.1 × 10^5^
*n*	0.92	0.95	0.93	0.93
*ε*_∞_	8.5	5.1	1.9	3.3
$f_{\max}^{\tan\delta }$/s^−1^	3000	3000	150	1050
(tan*δ*)_max_	5.8	10.0	12.9	4.7
*d*_0.5_/10^−4^ m	1.42	1.55	2.0	1.55
*λ*/m	1.3 × 10^−6^	3.5 × 10^−7^	2.8 × 10^−7^	1.5 × 10^−6^
*σ*_dc_/A(V m)^−1^	5.6 × 10^−5^	5.1 × 10^−5^	2.1 × 10^−6^	5.4 × 10^−6^
*f*_σ_/s^−1^	6.3 × 10^5^	3.6 × 10^5^	4.0 × 10^4^	5.9 × 10^4^
*DX*/m^2^ s^−1^	9.9 × 10^−14^	1.25 × 10^−14^	4.8 × 10^−16^	1.5 × 10^−15^
*c*/*c*_0_	1.2 × 10^−7^	7.3 × 10^−7^	6.5 × 10^−7^	3.1 × 10^−8^
*v*_drift_/m s^−1^	4.0	0.30	0.016	0.19
