# Supplementary material for: Low Frequency Dielectric Relaxation and Conductance of Solid Polymer Electrolytes with PEO and Blends of PEO and PMMA
Source: Polymers (Basel). 2020 Apr 27;12(5):1009. doi: 10.3390/polym12051009 (PMC7284942; doi:10.3390/polym12051009)
Supplement: Supplementary file 1 [file polymers-12-01009-s001.pdf]

Supplementary file

Low Frequency Dielectric Relaxation and Conductance of Solid Polymer Electrolytes with PEO and Blends of PEO and PMMA

Chin Han Chan * and Hans-Werner Kammer

Faculty of Applied Sciences, University Teknologi MARA, 40500 Shah Alam, Malaysia; hans-werner.kammer@jsk-medianet.de

* Correspondence: cchan_25@yahoo.com.sg

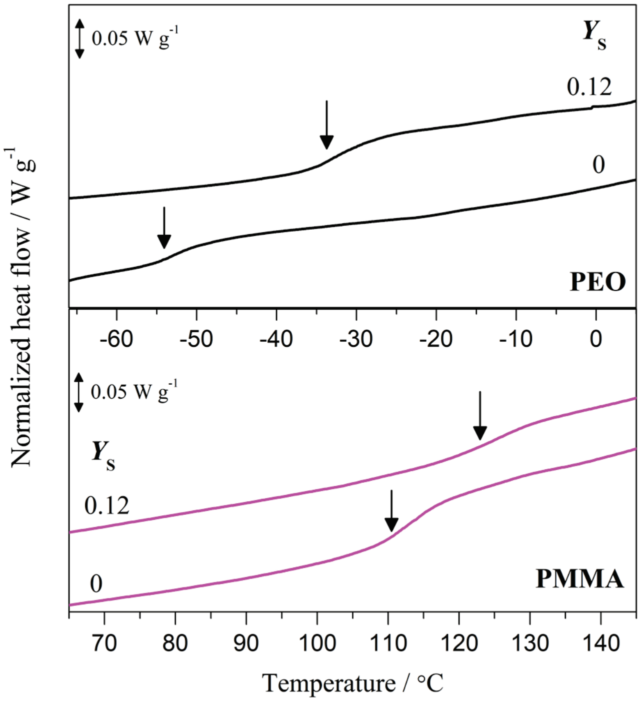

Figure S1. DSC scans for the two parent polymers versus added salt content Y_S. Arrows show the glass transition temperatures

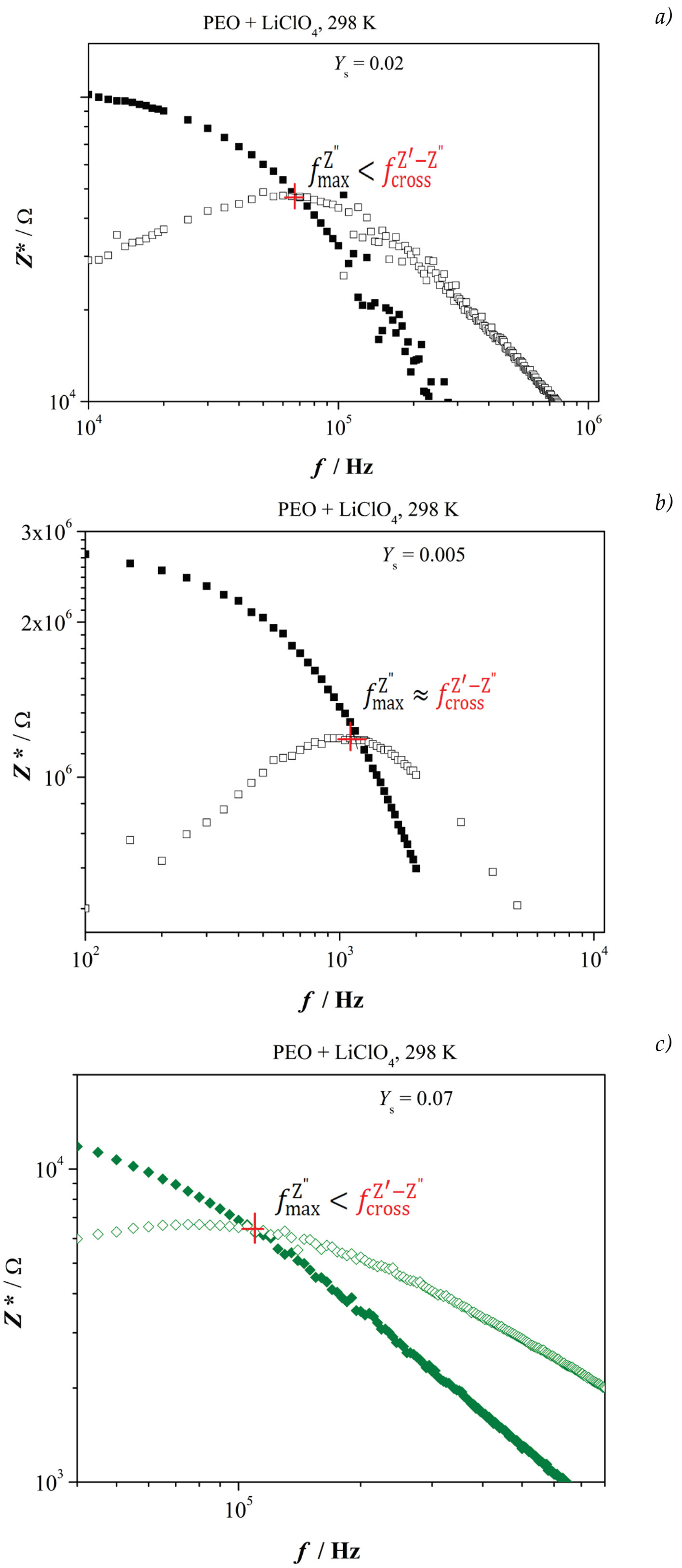

Figure S2. Impedance spectra of PEO with indicated salt concentration; Z''– open marker

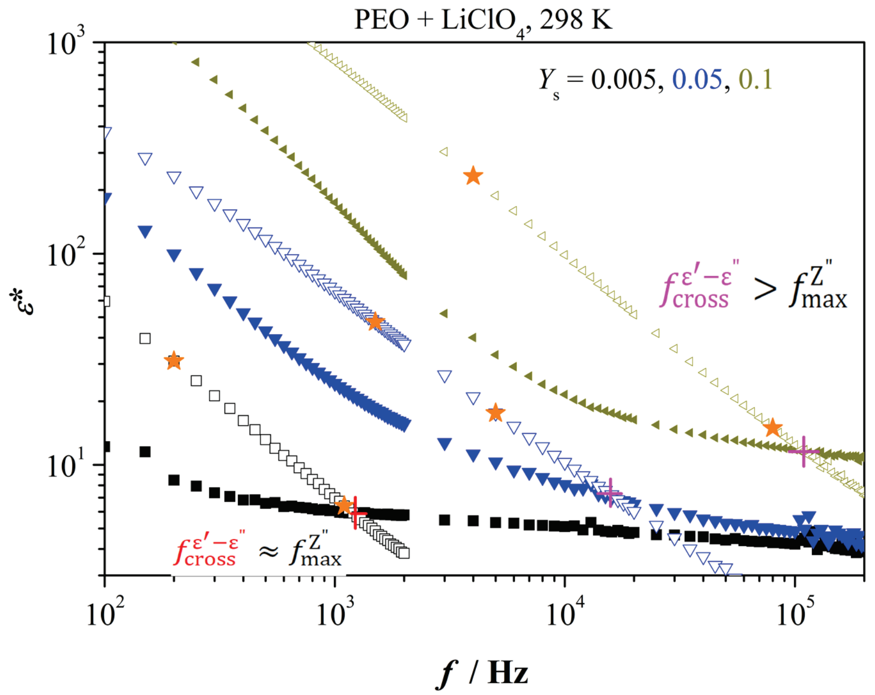

Figure S3. Selected permittivity spectra for the indicated salt concentration; ε' – solid marker; stars mark the low-frequency range $f_{min}^{Z''} \ldots f_{max}^{Z''}$
